# A Container for Storing and Transporting Bulk Material Samples

**DOI:** 10.3390/ma17235965

**Published:** 2024-12-05

**Authors:** Zbigniew Suchorab, Dagmara Olszewska-Pastuszak, Krzysztof Tabiś, Kamil Pluta

**Affiliations:** 1 Faculty of Environmental Engineering, Lublin University of Technology, ul. Nadbystrzycka 40B, 20-618 Lublin, Poland; 2 Aquapol Poland CPV Research Laboratory Krzysztof Tabiś, ul. Stefana Żeromskiego 12, 58-316 Świebodzice, Poland; dagmara.olszewska-pastuszak@drysenslab.pl (D.O.-P.); ktabis@aquapol.pl (K.T.); kamil.pluta@drysenslab.pl (K.P.)

**Keywords:** building materials, moisture, drying, transport, container

## Abstract

This article presents problems related to the storage of building material samples and discusses the related requirements and standards. Solutions for containers to store material samples were proposed and tests were performed in accordance with the EN ISO 12570 standard to demonstrate that all the water that may have condensed during the samples’ transport in the self-designed, closed container evaporates from the lid when unscrewed and placed under the container during the drying process. The aim of this study was to test the tightness of self-designed containers for transporting bulk samples. The drying efficiency at elevated temperatures in moisture tests for bulk material containers was determined and, finally, the influence of ambient conditions on a sample placed in a container for bulk material transport was estimated. The results confirmed that the designed container is vapour-tight and allows the collected material to be protected against evaporation during transport from the sampling site to the laboratory. Under extreme transport conditions, the water contained in the sample in a closed container partially evaporates from the material and condenses on the lid but this is taken into account when balancing the moisture of samples in the laboratory and does not falsify the readouts.

## 1. Introduction

The essence of laboratory testing is to obtain reliable results. In accordance with the guidelines of the ILAC (International Laboratory Accreditation Cooperation), testing laboratories must meet a number of requirements derived from international standards and testing methods to confirm their competence and provide reliable information, in accordance with ISO/IEC 17025:2017 [1]. Conducting reliable moisture testing of actual building structures is of particular importance, especially in historic and residential buildings. Moisture poses a threat to the sustainability of cultural assets as well as to the health of the occupants of damp buildings. The problem of moisture in historic buildings has been presented in numerous scientific publications [2,3,4,5]. On the other hand, M. Hodgson described the phenomenon of the sick building syndrome (SBS), i.e., health complaints occurring while living in damp residential buildings [6]; Flannigan et al. [7], on the other hand, highlighted the development of harmful microorganisms, which is determined by the presence of water in buildings. The phenomenon of dampness and the resulting consequences have been widely studied. In their article [8], Hall, C. and Hoff, W.D. described the transport of water in building materials that affects the structural properties of brick, stone, and concrete. V. Fassina et al. [9] highlighted the durability of plaster applied to the brick masonry of historic buildings exposed to moisture [10]. B. Lubelli et al. addressed the detrimental effects of salts transported by water deep into brick masonry [11]. The results of the tests allow the diagnosis of the causes of dampness and enable the proper selection of damp-proofing [12,13]. Samples, taken from actual buildings, must be representative and reflect the condition of the entire building. Testing consists of a sequence of interdependent stages which include sampling, storing, transporting and preparing for analyses, and performing the analyses to interpret the results [14]. All errors made during various stages are a component of the measurement error and affect the quality of the results. Acting in accordance with the organizations accrediting laboratories requires testing laboratories to estimate measurement uncertainty [15]. It is common practice to narrow down the uncertainty to the testing analysis itself, ignoring the uncertainty of collection, storage, and transport, the components that may be most significant in the total measurement uncertainty [16,17].

EN ISO 12570 [18], as applied to porous permeable materials, describes a general method for determining the water content of building materials by drying at elevated temperatures. The standard does not specify a sampling method but it does state that if sampling is carried out in the field or if drying cannot take place immediately after cutting the samples, the test samples should be packaged in vapour-proof metal containers to avoid any changes in moisture content before drying. In addition, the construction material of the specimen packaging including the seal must be non-corrosive and resistant to high temperatures.

ASTM D 2216 [19] also describes a method for determining the water content (moisture content) of bulk soils, rocks, and similar materials by drying at elevated temperatures. Section 7.1 of the standard states that the test material, prior to analysis in the laboratory, should be stored in an airtight, non-corrosive container, at a temperature between 3 and 30 °C, in an area that prevents direct contact with sunlight. In addition, care is taken to check that the lid is properly fitted to ensure that the container is airtight; this prevents the loss of water extracted from the material inside and prevents the absorption of moisture from the atmosphere after drying the sample and prior to the final determination of mass.

A separate parameter that should be taken into account when measuring real-world objects is the time that elapses between sampling and testing in the laboratory. The Environmental Protection Agency (EPA) has developed internal procedures for sample transport and storage [20]. These specify the maximum sample storage time and conditions during transport. The transport temperature should not exceed 6 °C, the sample delivery time to the laboratory should be 24 h, and the storage time in the laboratory should be 48 h.

This is outlined in more detail in the EPA guidelines [21], where procedures are described for both larger samples (core type) and smaller samples (40 mL glass vials), respectively: a storage time of 48 h at temperatures below 6 °C, and when frozen below −7 °C, this time is extended to 14 days.

In this article, attention is paid to the problem of storage and transport of samples for bulk material testing. A study was carried out on the feasibility of a container for testing materials taken for moisture analysis.

This article discusses issues related to the storage and transport of bulk samples of building materials. Problems related to the storage of samples of building materials are presented, the requirements related to them are discussed, and standards are listed. In the latest available literature, there is a lack of research on containers for the transport, storage, and testing of building materials, and the novelty in this work is the proposed model of a container for storing material samples and its usability in relation to testing in accordance with the EN ISO 12570 standard [18]. The goal is to undertake research that occurs during the accreditation process of a laboratory, in accordance with the international standard ISO/IEC 17025 [1]. As a part of research, laboratories carry out tests to determine the moisture content of materials by drying at an elevated temperature, and appropriate containers are necessary for storing and transporting samples. Commercially available containers do not guarantee vapour tightness and are susceptible to corrosion associated with chemical substances that are commonly found in building materials. It has also been noted that during the transport of samples, water that was extracted from the material and condensed inside the container on the lid disturbed the measurement result. In connection with this, the need for research on containers was noted, which has practical significance in ensuring the non-changeability of material samples taken from representative locations in buildings.

The aim of this study was to test the tightness of containers for storing samples at high temperatures, with repeated heating and cooling processes, and exposure to materials with different values of pH. The tightness of the container was checked using a tightness tester developed and manufactured according to our own idea (patent no.: 242123) [22]. Tests were also carried out in accordance with EN ISO 12570 [18] to show that all the water that could condense during the transport of the sample in a closed container evaporates when the lid is unscrewed and placed under the container during the drying process. The drying efficiency of the container was determined. In addition, in order to check the influence of environmental conditions on the sample placed in the container in relation to the moisture content in the tested material, a test was carried out in a climatic chamber.

## 2. Materials

### 2.1. A Kit for Measuring the Tightness of a Container

**Figure 1:**
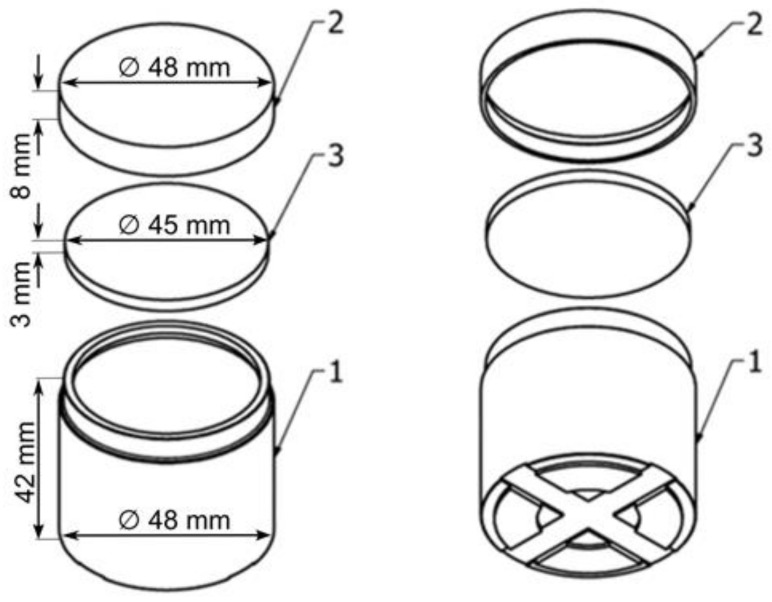
Scheme of a container for transporting bulk samples.

The subject of this research was a container for transporting loose samples (patent no.: 242114) [23], shown in Figure 1. The container was made of high-quality stainless steel, which reduces the impact of chemical and physical factors on its operational parameters and prevents corrosion. Each lid was individually adapted to a given container. There are identifying numbers on both the body and the lid. An additional measure for ensuring the tightness of the container is a silicone gasket, resistant to high temperatures, placed in the lid. By screwing the lid onto the body, the gasket provides an airtight sample storage environment. Due to the use of the container in gravimetric tests—the main element of which is drying the material in an open container—after placing the lid under the body, special grooves were cut on the bottom of the container; these enable evaporation of the water extracted from the sample that condenses on the lid, allowing real weight loss results to be obtained.

**Figure 2:**
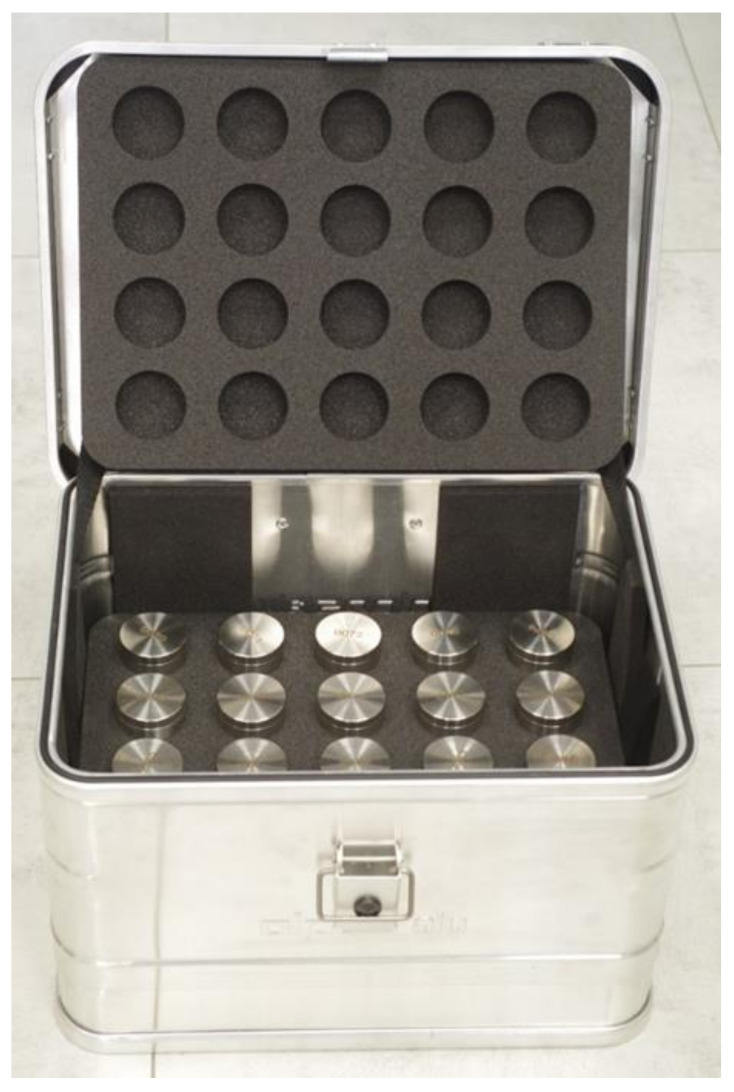
Photograph of the container for transporting bulk samples.

For the duration of the transport, the containers described above were placed in a specially designed metal transport box (Figure 2), inside which there were three levels of protective sponges, with holes for 60 containers.

**Figure 3:**
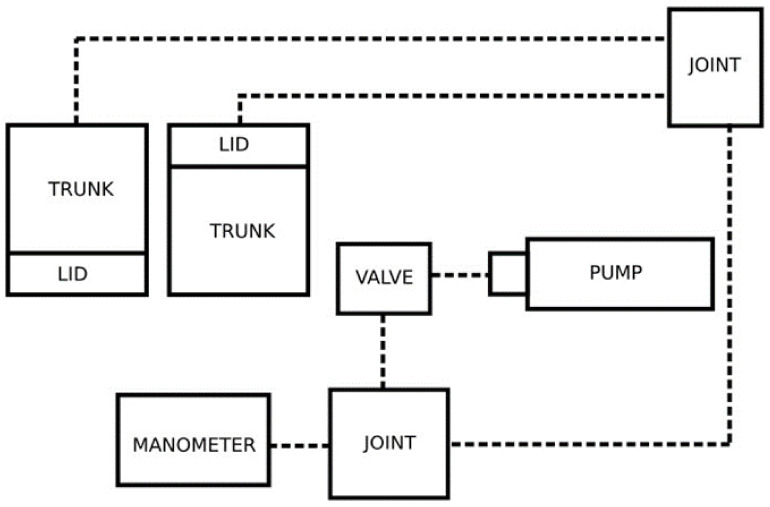
Schematic of the device for testing container tightness.

The tightness of the containers was checked using a specialised device (patent no.: 242123) [22], shown in Figure 3. The measuring equipment consisted of a pump generating overpressure, a manometer showing the pressure difference between the ambient pressure and the pressure inside the tested container, valves cutting off the air supply, and ducts supplying air to the tested containers.

### 2.2. Laboratory Kit for Determining the Drying Efficiency of the Container in Which Samples Were Transported

**Figure 4:**
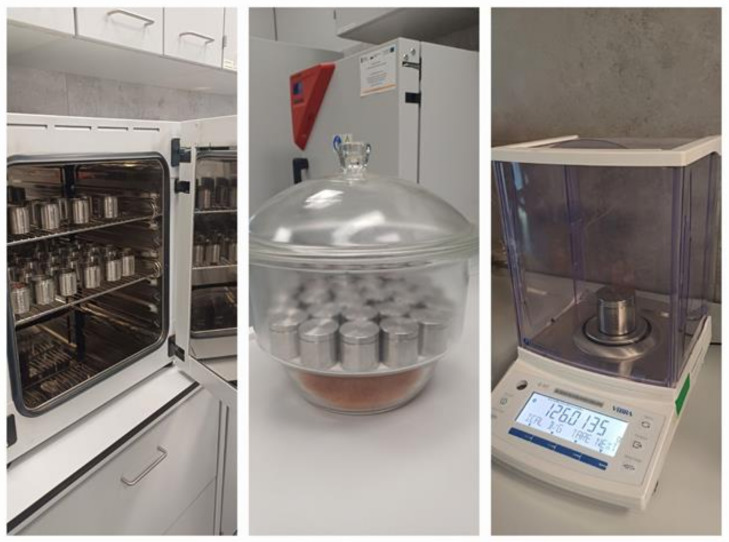
Laboratory kit for determining the drying efficiency of the container in which samples were transported.

Analyses of the evaporation of water—that could condense during the transport of the sample in a closed container from the lid, which was unscrewed and placed under the container during the drying process—were carried out in accordance with the EN ISO 12570 standard [18]. For mass measurements, a calibrated HT224RCEN scale (VIBRA SHINKO DENSHI CO., Tokyo, Japan) was used, with a measurement accuracy of up to 0.0001 g. In order to reproduce the most unfavourable conditions for transporting samples from the collection site to the laboratory (temperature of approx. 50 °C), a FED56 dryer was applied (BINDER GmbH, Tuttlingen, Germany). The dryer was also used to dry samples and containers to a constant mass, taking into account the mass of the body and cap separately. A desiccator filled with silica gel was also used for testing. The laboratory set-up for determining the drying efficiency of the container in which the samples were transported is shown in Figure 4.

The samples for the above-mentioned analysis were prepared from solid ceramic bricks, class 15. The moisture percentage was selected taking into account the possible spectrum of the tested material. The characteristics of the experiment required the use of samples with moisture states > 5%, in a growing system, taking into account the amount of water extracted from the samples under the influence of increased temperatures. Bricks from one batch were crushed and completely dried. Table 1 shows the ratio of the mass of the material to the mass of added water [g] in the analysed samples and the assumed mass moisture of the material [%]. Deionised water with conductivity below 1 µS/cm was used. Seven different moisture variants of samples were prepared, ranging from 5.3% to 25%. For statistical purposes, each moisture variant was prepared in five repetitions, which required using thirty-five containers in total. The values in Table 1 are the calculated and determined amounts of water and material needed to obtain the desired mass moisture content. The assumed percentage mass moisture content, calculated relative to the dry material, is used in the research part of this article.

### 2.3. Set for Testing in a Climatic Chamber

In parallel, tests were carried out to explore the impact of environmental conditions on the samples sealed in containers. For this purpose, a set of samples was prepared for testing in a climatic chamber. Crushed, porous material with a fraction above 4 mm, which was solid ceramic brick from the same source, was placed in a laboratory dryer at a temperature of 200 °C. Then, the calcined material was transferred to 60 containers, 10 containers for each moisture value, presented in Table 2.

The mass moisture of the samples presented in Table 2 was selected so that the samples represented the division consistent with the literature data [24,25,26], where, due to the moisture content of the walls, *W_m_*—mass moisture [%] is within the following ranges:

- *W_m_* = 0–3%—walls with permissible moisture;
- *W_m_* = 3–5%—walls with increased moisture;
- *W_m_* = 5–8%—moderately damp walls;
- *W_m_* = 8–12%—very damp walls;
- *W_m_* > 12%—wet walls added.

In order to prepare samples with a moisture content of 0%, 2%, 4%, 6%, 10%, and 13%, deionised water with a conductivity below 1 µS/cm was added to the completely dried material. The selected amount of material and water is presented in Table 2.

As in the case of drying efficiency tests, the moisture content of samples was analysed according to EN ISO 12570 [18] and was carried out in a BINDER laboratory dryer. Weight measurements were conducted on a VIBRA laboratory scale. Variable environmental conditions were obtained in a calibrated MKF115 climatic chamber (BINDER GmbH, Tuttlingen, Germany).

## 3. Methods

### 3.1. Tightness Testing of a Container for Transporting Bulk Samples

Acting in accordance with the guidelines of the organization accrediting research laboratories and in order to meet the requirements of the EN ISO 12570 standard [18], a container for transporting bulk samples was developed and manufactured (patent no.: 242114) [23]. Containers numbered 0001 to 0118 were tested to confirm their tightness and usability for gravimetric tests.

The schematic view of the measuring station, as shown in Figure 3, made it possible to measure the tightness, taking into account both the cover and the container for storing bulk materials. For this purpose, pipes were connected to them from a pump, which created an overpressure to such a level that the pressure difference between the ambient pressure and the pressure inside the tested container was 1 bar. After obtaining the determined pressure values, the valve was closed and the air supply was cut off. The containers were left in this arrangement for 24 h. After 24 h, the value from the manometer was read to check whether the pressure difference had changed. The test was repeated five times for each container.

### 3.2. Determining the Effectiveness of Drying at Elevated Temperatures in Moisture Tests for Containers Transporting Bulk Materials

The gravimetric method (also described as the weighing and drying method or the Darr method) is considered the most reliable method for determining the moisture content in building materials [24,25]. The results obtained using this method are references to the results obtained using other methods. For fragile materials, where loss during unpacking is possible, the EN ISO 12570 [18] standard recommends a testing procedure consisting of taking material samples, placing them in tight containers, and delivering them to the laboratory. Then, the first mass measurement of the sample is made in a closed container, and the material in the open container is dried together with the entire packaging in a laboratory dryer at a temperature of 105 °C to a constant weight. The final step is to separate the sample from the packaging and measure the weight of the container. Calculations of the mass moisture content of the tested material are carried out according to Formula (1):

*W_m_* = (*m* − *m*_0_ − *m_p_*)/*m*_0_, (1)

where

*W_m_*—mass moisture [kg/kg];
*m*—weight of the test sample with packaging before drying [kg];
*m*_0_—mass of the test sample after drying [kg];
*m_p_*—weight of the packaging after drying [kg].

The result is determined by the loss of mass of the sample placed in the container under the influence of increased temperature. The purpose of the analysis is to verify the usefulness of the container by confirming that all the water extracted from the sample and condensed on the lid evaporates, allowing the actual weight loss results to be obtained.

The building material prepared in accordance with Table 1, in five repetitions, was placed in thirty-five containers. Closed containers with the material were stored at 50 °C for 24 h in order to recreate extreme conditions in which the sample can be transported. After extracting water from the sample and condensing it on the lid, the lid and the body of the container with the sample were weighed separately and the procedures were followed in accordance with the guidelines of the EN ISO 12570 standard [18].

The lid with a seal was placed under the matching body. In this arrangement, the sample was placed in a laboratory dryer and dried at a temperature of 105 ± 2 °C for 24 h until constant mass was achieved (constant mass is achieved when the change in mass between three subsequent weighings in a 24-h period is less than 0.1% of the total weight). The samples were cooled in a desiccator between weighings and weighed in a closed container after reaching a temperature of 30 °C to 40 °C. To demonstrate the evaporation of water from the lid, the lid and the container without the lid were weighed separately. The EN ISO 12570 standard [18] recommends the use of units expressed in kg, however, due to the weight of the samples, the results are presented in g and converted to the percentage of mass moisture of the material. Assuming the accuracy of the equipment used is in accordance with the standard and allowing for an additional 1% moisture error due to sample processing, the mass moisture accuracy is estimated as within 3%.

### 3.3. Determining the Influence of Ambient Conditions on a Sample Placed in a Container for Transporting Bulk Materials in Relation to the Tested Materials’ Moisture Parameter

The aim of the analysis was to demonstrate that the mass moisture of the material placed in a tight container does not change under the influence of environmental conditions to which the samples are exposed during transport.

The tests were carried out in a climatic chamber with an assumed relative air humidity of 85% and variable temperature conditions in the following sequence of 40 °C, 30 °C, 20 °C, and 0 °C, at weekly intervals. The relative humidity was selected based on the ÖNORM B 3355:2017 standard [27], which provides conditions for testing hygroscopic moisture in building materials.

Samples with the given moisture, in accordance with Table 2, were stored in closed containers, at a constant temperature, for a period of 7 days. The containers were weighed regularly to monitor the change in their mass and to check whether they had achieved equilibrium with the environment (constant mass). The containers were divided into two groups: The first group of 30 containers, 5 samples for each moisture content, was controlled by daily measurement of their weight for a period of 26 days at the aforementioned temperatures. These containers remained closed throughout the study period under established environmental conditions. In the case of the second group of 30 containers, the material was subjected to gravimetric analysis in accordance with the EN ISO 12570 standard [18]. Five samples for each moisture were tested at weekly intervals. In this way, it was determined whether there were changes in the moisture content of the material under the influence of the environmental conditions established in the climatic chamber. Testing the water content in building materials in accordance with the EN ISO 12570 standard [18] requires drying at an elevated temperature; therefore, for the second group of containers, the material was prepared again (in accordance with Table 2) after each week at the given temperatures.

## 4. Results and Discussion

### 4.1. Results of Tightness Testing of a Container for Transporting Bulk Samples

The manometer reading, checked every 24 h for 5 days in five repetitions for 118 containers, remained unchanged. The set pressure difference of 1 bar was maintained on each day of the study. In this way, the tightness of the container was confirmed in accordance with the requirements of the EN ISO 12570 standard [18] and the requirements of the ASTM D 2216 standard [19] regarding the proper fit of the lid to the body, which is intended to protect against the loss of water extracted from the material placed inside and protects against moisture absorption from the atmosphere after drying the sample and before the final mass determination [18,19]. The results indicate that the storage time of samples in the laboratory, with constant environmental parameters, may be 5 days compared to 48 h recommended by the US Environmental Protection Agency [20].

### 4.2. Results of Determining Drying Efficiency

This subchapter presents the results of testing the effectiveness of drying the material and the container at elevated temperatures.

#### 4.2.1. The Result of Testing the Efficiency of the Material Drying at Elevated Temperatures

**Figure 5:**
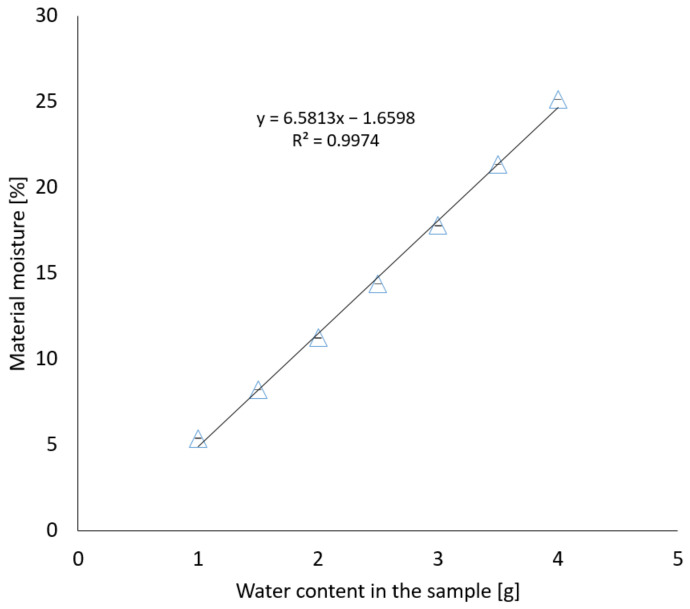
Dependence of material moisture on water mass.

Figure 5 shows the dependence of the average mass moisture content of the material from five repetitions for each moisture variant on the mass of applied water in accordance with Table 1.

The mass moisture of the samples after testing in accordance with the EN ISO 12570 standard [18] and taking into account the accuracy of the method is consistent with that assumed in Table 1. The variability of the material moisture in relation to the applied water is linear, and the value of the determination coefficient R^2^ is over 99%, which indicates a good fit of the linear model to the examined relationship.

#### 4.2.2. Results of Testing the Efficiency of the Container Drying at Elevated Temperatures

**Figure 6:**
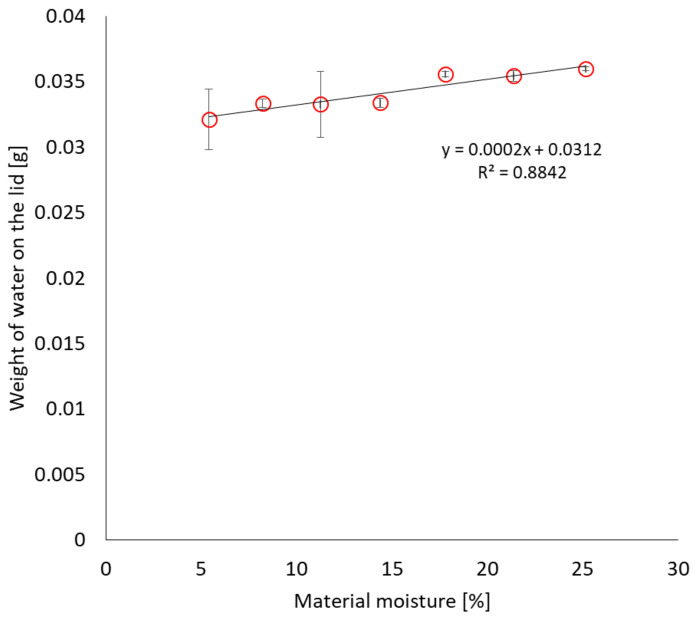
Dependence of the mass of water condensed on the lid, after storage under extreme transport conditions, on the material moisture.

Additionally, it was tested whether the design of the container allows for effective drying and provides reliable results of the moisture content of the bulk material. When reproducing the sample transport conditions at 50 °C in a closed container, water extracted from the building material condensed on the lid in small amounts compared to the total moisture of the sample. At a material moisture of 5.4%, the mass of water on the lid was on average 0.032 g. As the sample moisture increased, the amount of water on the lid increased to a maximum level of 0.036 g in the case of a maximally saturated sample, as shown in Figure 6 Similarly to the previous case, the tested relationship is linear and is characterised by a relatively high value of the coefficient of determination R^2^, greater than 88%.

**Figure 7:**
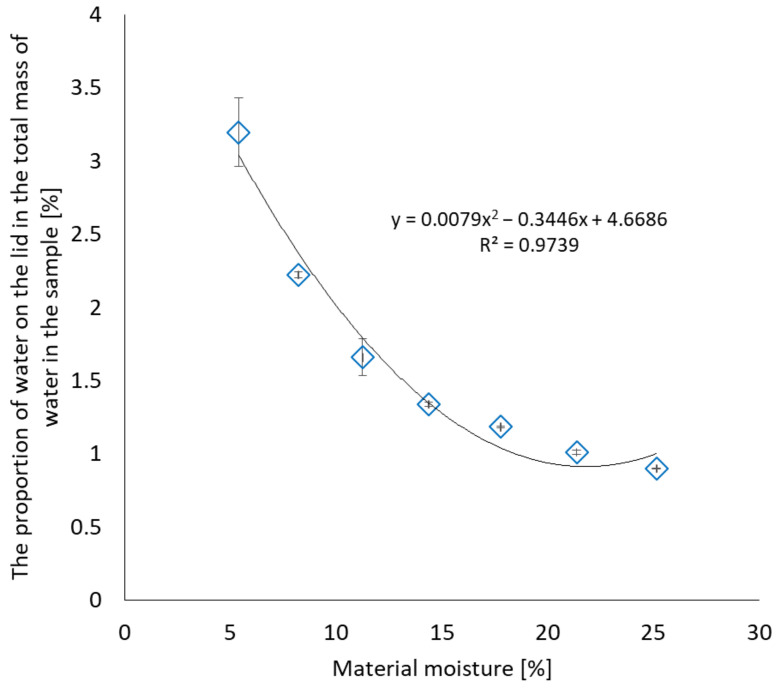
Dependence of the percentage of water condensed on the lid on the percentage of moisture in the material.

Figure 7 compares the mass moisture of the material with the percentage of water condensed on the lid in the total mass of water in the sample. With the lowest sample moisture content, the percentage share was the highest and accounted for 3.2% of the total moisture content; in comparison, when the material moisture exceeded 25%, it was only 0.9%.

The mass of the container (taking into account the lid and the container without the lid separately) after the test, i.e., drying to constant weight, determining the moisture content of the material, and separating the sample from the container, returned to the state before the analysis. The maximum difference was 0.0009 g, which is a negligible value within the error range of the equipment used. This means that all the moisture has evaporated and the grooves cut into the container body fulfil their function.

### 4.3. Results of Measurements of the Influence of the Environment on the Sample in the Container

**Figure 8:**
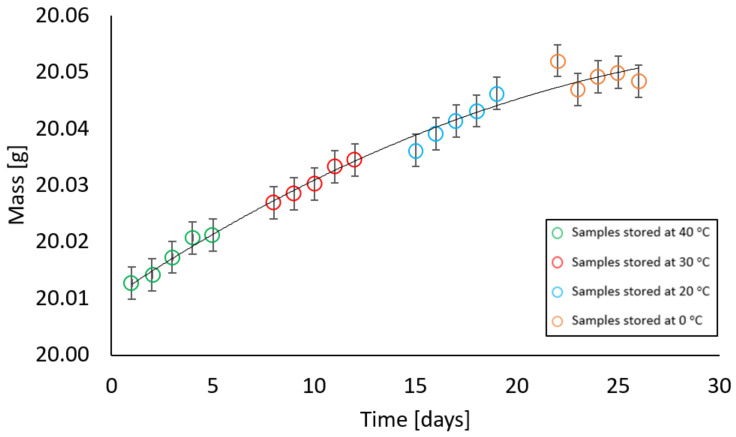
Dependence of the mass of a sample with a mass moisture of 0% on the storage time in a climatic chamber at a relative humidity of 85% with weekly intervals at temperatures of 40 °C, 30 °C, 20 °C, 0 °C (max error is 0.008 g).

Samples with zero moisture gradually increased their mass throughout the entire period of stay in the climatic chamber at 85% relative humidity at the following temperature sequence: 40 °C, 30 °C, 20 °C, and 0 °C. The test results are presented in Figure 8. It was observed that the mass increase after 5 days at 40 °C was 0.0085 g, and after 26 days it was 0.0357 g. The maximum increase was 0.2% of the total initial mass of the sample.

**Figure 9:**
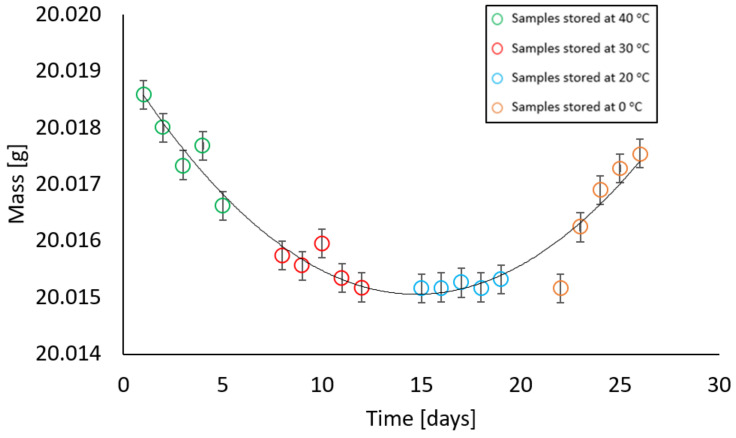
Dependence of the mass of a sample with a mass moisture of 2% on the storage time in a climatic chamber at a relative humidity of 85% with weekly intervals at temperatures of 40 °C, 30 °C, 20 °C, 0 °C (max error is 0.006 g).

**Figure 10:**
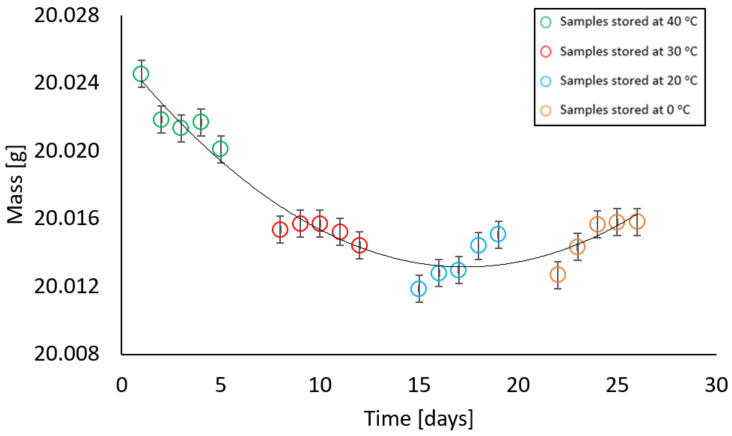
Dependence of the mass of a sample with a mass moisture of 4% on the storage time in a climatic chamber at a relative humidity of 85% with weekly intervals at temperatures of 40 °C, 30 °C, 20 °C, 0 °C (max error is 0.013 g).

**Figure 11:**
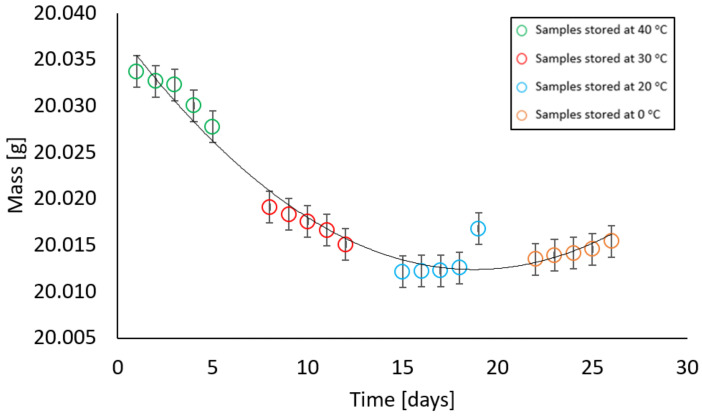
Dependence of the mass of a sample with a mass moisture of 6% on the storage time in a climatic chamber at a relative humidity of 85% with weekly intervals at temperatures of 40 °C, 30 °C, 20 °C, 0 °C (max error is 0.009 g).

**Figure 12:**
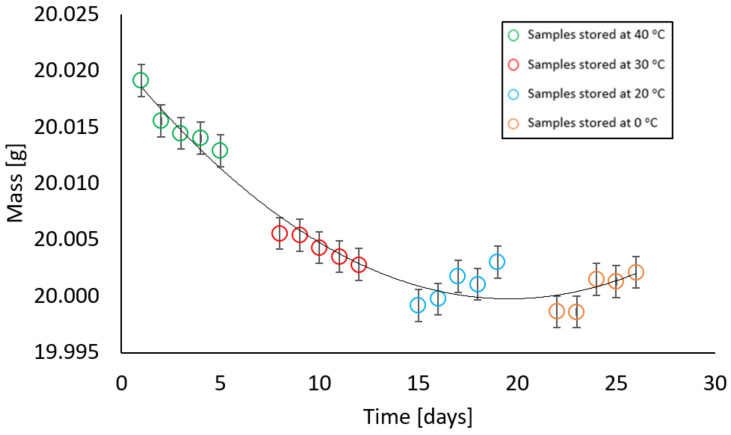
Dependence of the mass of a sample with a mass moisture of 10% on the storage time in a climatic chamber at a relative humidity of 85% with weekly intervals at temperatures of 40 °C, 30 °C, 20 °C, 0 °C (max error is 0.0042 g).

**Figure 13:**
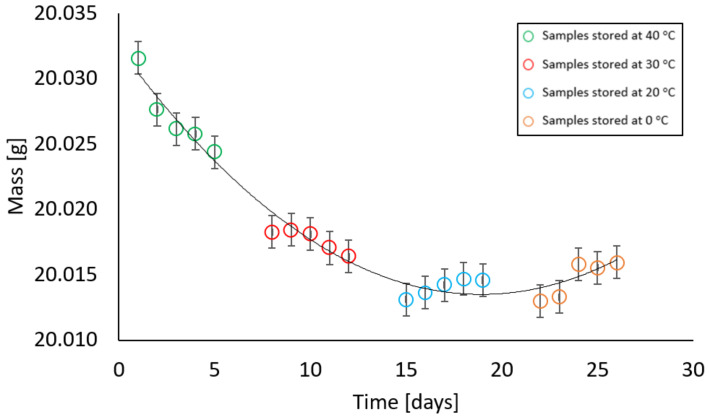
Dependence of the mass of a sample with a mass moisture of 13% on the storage time in a climatic chamber at a relative humidity of 85% with weekly intervals at temperatures of 40 °C, 30 °C, 20 °C, 0 °C (max error is 0.01 g).

In contrast, samples with a moisture content different from zero behaved in such a way that at the first stage of measurement, there was a decrease and then an increase in their mass. All samples, i.e., with moisture contents of 2%, 4%, 6%, 10%, and 13% showed a similar relationship at given temperatures. At temperatures of 40 °C and 30 °C, a decrease in the weight of the samples was recorded, at a temperature of 20 °C the weight remained constant, while an increase in weight was noted at a temperature of 0 °C. The relationships above are illustrated in Figure 9, Figure 10, Figure 11, Figure 12 and Figure 13.

The maximum weight loss of samples with an initial moisture content of 2% was 0.0034 g and occurred on the 12th day of the test, which corresponded to being kept in a climatic chamber for a sequence of a week at 40 °C and 5 days at 30 °C; however, after 26 days, taking into account the final stage at 0 °C with 85% relative humidity, the sample increased its mass in such a way that the difference between the initial and final mass was only 0.001 g.

In samples with an initial moisture content of 4%, after being kept at 40 °C and 30 °C, the mass decreased by 0.0127 g, while after 26 days the difference was 0.0087 g.

At a mass moisture of 6%, the samples were dried by a maximum of 0.0216 g, while after being held at 20 °C and 0 °C, the difference in mass from the initial weight was 0.0183 g. Similarly, in samples with a moisture content of 10% and 13%, the difference did not exceed 0.02 g.

In order to determine the significance of the influence of the independent variable—which is the time of storing samples in the climatic chamber in weekly intervals at temperatures of 40 °C, 30 °C, 20 °C, and 0 °C on the dependent variable, i.e., the mass of the sample stored in the climatic chamber—regression models of the polynomial type and analysis of variance (ANOVA) were performed. Table 3 shows the results of the fit measures, which explain the statistical significance and significant proportions of variance.

All obtained results are characterised by good-fitting parameters. The R^2^ ranges between the values [93.4%; 98.5%]. As a result of the analysis, a statistically significant effect of the variable was obtained, which was the time of storing the samples in the climatic chamber at temperatures of 40 °C, 30 °C, 20 °C, and 0 °C. The comparisons showed statistically significant differences between all groups (*p* < 0.001). Power estimators for all equations are expressed in the form (^−05^x^2^), which indicates that the values are equally close to the set. All intercepts in the equations oscillate around 20. The graphs show that when samples are kept, regardless of their moisture variant, their mass stabilises around 20 °C; however, the change in the value of the resulting quantity is very small and may result mainly from the range of measurement error.

**Figure 14:**
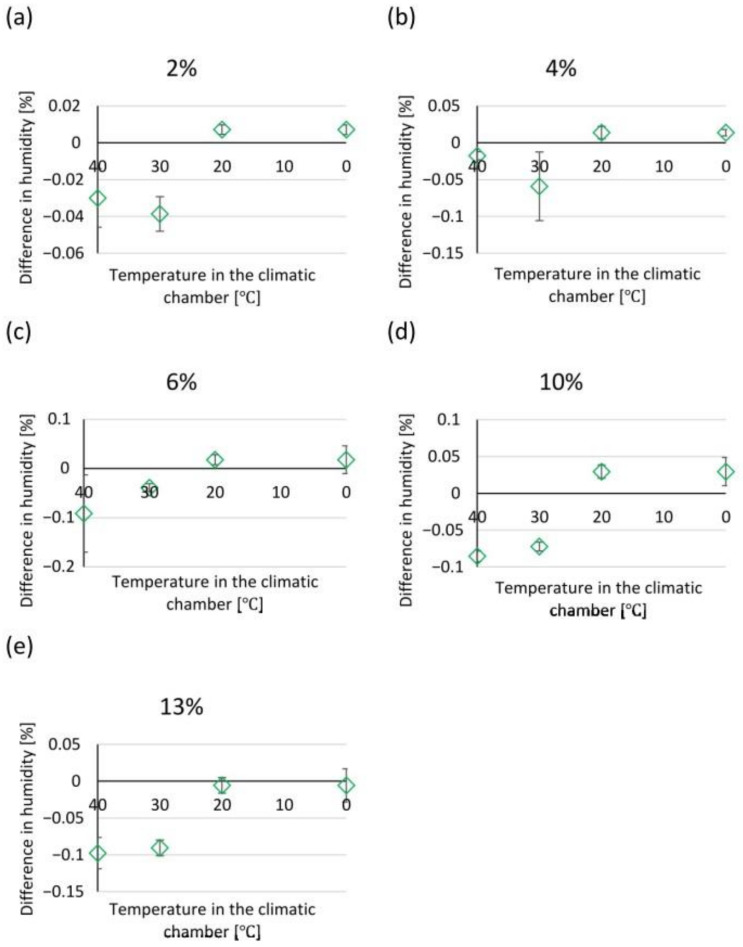
Dependence of the difference in moisture on the temperature at which a sample with given moisture parameters was stored. (**a**) samples with 2% mass moisture content, (**b**) samples with 4% mass moisture content, (**c**) samples with 6% mass moisture content, (**d**) samples with 10% mass moisture content, (**e**) samples with 13% mass moisture content.

The experimental results illustrate how moisture is absorbed and released from the air. Hygroscopic moisture of a material is the share of total moisture that appears when water is taken in accordance with the relative air humidity and ambient temperature and an equilibrium state is achieved under given conditions [24]. Most often, the ability to absorb and release moisture is presented using the so-called sorption isotherm curves, which show the moisture content in the material at relative humidity from 0% to approximately 95% [28]. The type of material has a significant influence on the sorption moisture because the hygroscopic moisture associated with condensation depends largely on the radius of the capillary pores. On the basis of the literature data, it was found that the formation of capillary condensation in solid bricks fired from clay begins at a relative humidity above 80% [29]; therefore, in accordance with the ÖNORM B 3355:2017 standard [27], tests were carried out at a relative humidity of 85% (±5%). It was noticed that at temperatures of 40 °C and 30 °C the material released moisture but only to a small extent. The maximum difference between a sample with a given moisture content before being placed in the chamber and after being kept in the chamber was 0.0216 g, which for samples weighing 20.03 g was 0.004% of their mass. However, at a temperature of 20 °C—which is within the range that, according to the ASTM D 2216 standard [19], the research material should be transported in whilst held inside an airtight, non-corrosive container before analysis in the laboratory—the values remained constant. The largest noticeable difference at a given temperature was 0.003 g. The increase in mass at 0 °C is justified by the water vapour condensation. Cooled air with a given water vapour content reaches a state of saturation. In this study, although the mass measurement was carried out immediately after removing the container from the climatic chamber, condensation on the surface of the container was noticeable and increased the mass of the sample. The increase in weight was not due to the material absorbing moisture but due to moisture on the container. The overall mass increase in this range was from 0.0019 g to 0.0036 g. Values in this range do not significantly affect the tested moisture parameter of the tested material placed in the container. This is confirmed by the gravimetric measurement of the second group of samples, where the material was subjected to gravimetric analysis in accordance with the EN ISO 12570 standard [18]. The difference in material moisture content before and after being placed in the climatic chamber was calculated at weekly intervals at temperatures of 40 °C, 30 °C, 20 °C, and 0 °C with a relative humidity of 85%. The dependence of the difference in moisture before and after being placed in the chamber on the temperature at which the sample, with given moisture parameters, was stored is shown in Figure 14a–e.

The smallest differences in moisture, at a constant level, were noticed after keeping the samples at 0 °C and 20 °C, which confirms the hypothesis that the sample does not absorb water from the environment and the increase in mass in the first group of samples depended on the condensation on the surface of the container. The maximum moisture differences were observed in samples with the highest set moisture of 13%. After storage at 40 °C the difference was 0.1%, while at 30 °C it was 0.09%.

Taking into account the measurement accuracy resulting from the EN ISO 12570 standard [18], the significance of the differences shown is irrelevant to the test result. Therefore, it can be concluded that the container meets the standard requirement, which is to pack samples in vapour-tight metal containers.

Comparing our results to those from the world literature, it should be noted that there is no research on containers for the transport and storage of building materials in relation to testing the mass moisture parameter. However, the Food and Agriculture Organization of the United Nations has issued recommendations on the procedure for measuring soil moisture using the gravimetric method [30]. Point seven outlines that the sample should be stored in a clearly described, airtight container that does not allow moisture to enter or contaminate the sample. Additionally, before determining the moisture content, the soil should be stored in a tight container, in a shaded place, at a temperature of 3 °C to 30 °C. This confirms the above-mentioned assumptions of the ASTM D 2216 [19] standard regarding determining the water content in soils, rocks, and similar materials by drying at elevated temperatures. There are no publications of scientific studies confirming the assumptions indicated in the above standards and guidelines, hence the results presented in this article constitute added value in relation to moisture testing by drying at elevated temperatures. In our research, containers available on the market were used in testing the mass moisture of building materials by drying at an elevated temperature. The results were unreliable, the differences for the materials with a mass moisture content above 5% were on average 2%, while for materials with a mass moisture content above 9%, the differences were on average 4%. Due to the lack of tightness of the container, the results were always underestimated; additionally, corrosion of the containers was observed. John H. Zimmerman and Brian A. Schumacher [31] in their article proposed two containers for storing soils contaminated with volatile organic compounds and confirmed that they meet the EPA [21] guidelines for the storage time for 48 h. In this article, it was indicated that there were no significant differences in the results of mass moisture of samples stored for a week (confirmed at different temperatures). Therefore, the proposed time of 48 h—using the tested containers—could be extended. Lewand L. R. and Koehler D. from Neta World Journal [32] conducted research to determine which types of containers are best suited for maintaining the quality of the sample for the analysis of water content in insulated liquids. They tested glass bottles, glass syringes, and aluminium bottles. Transformer oil samples were stored in containers under the conditions of room temperature (approximately 22.5 °C) and high humidity (83% to 88%). After 8 weeks, it was checked how much in mg/km (ppm) the samples stored in the containers differed. In each variant of container, the sample absorbed moisture from the air but the amount was small, even lower than that specified in this article. Samples in glass bottles gained 0.00171%, glass syringes 0.0008%, while aluminium bottles, characterised by the best tightness, 0.00071%. However, the research methodology for determining the moisture content of the samples did not require drying at an elevated temperature (the Karl Fischer titration method was used); hence, there is no information about the use of the containers at high temperatures. In connection with the above, the containers could not be used in the research by drying at elevated temperatures.

## 5. Conclusions

The use of a vapour-tight container for transporting and storing samples for testing bulk materials is an inherent component of the work of an accredited testing laboratory that conducts tests in accordance with the EN ISO 12570 standard [18] as well as international standards dealing with the issues of sample storage and transport.

It has been shown that the researched container is vapour-tight and allows the collected material to be protected against evaporation during transport from the testing site to the laboratory. Once unscrewed, the container, together with the cap and seal, can be dried in an oven and its structure facilitates complete drying.

Under extreme transport conditions (50 °C), the water contained in the sample in a closed container partially evaporates from the material and condenses on the lid. The presence of water on the packaging is taken into account when balancing the moisture of samples in the laboratory.

Water on the lid in samples with a mass moisture content of approximately 5% constitutes 3.2% of the total result. During drying of both the material and the packaging, owing to the grooves present on the lower body of the container, the water from the lid evaporates during the determination of moisture using the drying method and is included in the result.

At high ambient relative humidity, a change in the mass of the sample in the container is observed as the temperature decreases. The dry material is characterised by a tendency to a slight, gradual increase in mass when kept in a climatic chamber at 85% moisture. However, the material with a given moisture, regardless of the percentage of mass moisture, shows a tendency to lose weight at temperatures of 40 °C and 30 °C. At 20 °C, the mass stabilises, which confirms that this is the temperature recommended for transporting the material to the laboratory. The increase in mass at 0 °C results from the condensation of water vapour on the surface of the container and does not affect the moisture of the material. Despite showing trends, the differences in the measured values are small and have no significance for the test result. High ambient moisture has no significant effect on the sample placed in the container.

Due to the lack of containers on the market dedicated to the storage and transport of samples of building materials that would meet the requirements of the PN-EN ISO 12570 [18] standard, it is recommended to use the containers tested in this article. Especially in accredited research laboratories, whose most important task is to obtain reliable results, the error resulting from the transport and storage of samples should be eliminated. The limitation of this article is that the research only considered one matrix–ceramic brick. Future research plans include checking the usability of containers on other research matrices such as wood, silicate, concrete blocks, or natural stone, which are characterised by different porosities and therefore have different water absorption models.

## 6. Patents

Patents resulting from the work reported in this manuscript are as follows: [22,23].

## Figures and Tables

**Table 1:** Pre-set material moisture values depending on the amount of water.

Sample Number	Ratio of Material Mass to Water Mass [g]	Mass Moisture [%]
1	19/1	5.3
2	18.5/1.5	8.1
3	18/2	11.1
4	17.5/2.5	14.3
5	17/3	17.6
6	16.5/3.5	21.2
7	16/4	25.0

**Table 2:** Ratio of material content to water amount.

Sample Mass Moisture [%]	Material [g]	Water [g]
0	20	0
2	20	0.4
4	20	0.8
6	20	1.2
10	20	2
13	20	2.6

**Table 3:** Pre-set material moisture values depending on the amount of water.

	Equation	R^2^	R^2^ (adj.)	F	*p*
0%	y = −3 × 10^−05^x^2^ + 0.0024 × x + 20.01	0.985	0.983	545.54	<0.001
2%	y = 2 × 10^−05^x^2^ − 0.0005 × x + 20.019	0.934	0.927	121.15	<0.001
4%	y = 4 × 10^−05^x^2^ − 0.0014 × x + 20.026	0.936	0.929	124.38	<0.001
6%	y = 7 × 10^−05^x^2^ − 0.0027 × x + 20.038	0.965	0.960	231.16	<0.001
10%	y = 5 × 10^−05^x^2^ − 0.0021 × x + 20.021	0.958	0.954	195.86	<0.001
13%	y = 5 × 10^−05^x^2^ − 0.002 × x + 20.032	0.977	0.974	355.32	<0.001

## Data Availability

The original contributions presented in the study are included in the article, further inquiries can be directed to the corresponding author.
